# Effective Medium Method for Chloride Diffusion Coefficient of Mature Fly Ash Cement Paste

**DOI:** 10.3390/ma12050811

**Published:** 2019-03-08

**Authors:** Hong Zhou, Xin-Zhu Zhou, Jian Zhang, Jian-Jun Zheng

**Affiliations:** 1 Architectural Engineering School, Jinhua Polytechnic, Jinhua 321007, China; 20050267@jhc.edu.cn; 2 School of Civil Engineering and Architecture, Zhejiang University of Technology, Hangzhou 310023, China; jzhang777777@163.com (J.Z.); jjzheng@zjut.edu.cn (J.-J.Z.)

**Keywords:** fly ash cement paste, chloride diffusion coefficient, effective medium approach, percolation theory

## Abstract

The chloride diffusion coefficient of concrete plays an essential role in the durability assessment and design of concrete structures built in chloride-laden environments. The purpose of this paper is to present an effective medium method (EMM) for evaluating the chloride diffusion coefficient of mature fly ash cement paste. In this method, a numerical method is used to estimate the degrees of hydration of cement and fly ash. Fly ash cement paste is then modeled as a two-phase composite material, composed of a solid phase and a pore space. By introducing the percolation theory, the EMM is modified to derive the chloride diffusion coefficient of fly ash cement paste in an analytical manner. To verify the EMM, a chloride diffusion test of fly ash cement paste at a curing age of up to 540 days is conducted. It is shown that, within a reasonable fly ash content, a larger fly ash content and/or curing age results in a smaller chloride diffusion coefficient. The chloride diffusion coefficient decreases with a decreasing water/binder ratio. Finally, the validity of the EMM is verified with experimental results.

## 1. Introduction

For concrete structures located in chloride-laden environments, the attack of chloride ions becomes the primary cause for the corrosion of reinforcements [1,2,3]. The penetration of chloride ions into concrete is controlled, to a large extent, by the chloride diffusion coefficient and the chloride binding of cement paste [4,5]. To reduce the chloride diffusion coefficient, various mineral admixtures are usually added to concrete. Fly ash is a byproduct resulted from coal power stations. Chemically, it contains 60–65% of SiO_2_, 25–30% of Al_2_O_3_, and 6–15% of Fe_2_O_3_. Such a special chemical composition makes fly ash exhibit a strong pozzolanic reactivity and improves the microstructure of cement paste particularly after 28 days of curing [6,7,8,9]. At a mesoscopic level, concrete is composed of bulk cement paste, interfacial transition zones (ITZs), and aggregates. Essentially, the ITZ is a cement paste with a high water/binder ratio. Compared with the bulk cement paste and ITZ, aggregates are almost impermeable to chloride ions. When the chloride diffusion coefficients of cement pastes with various water/binder ratios are determined, that of concrete can be immediately formulated with a three-phase composite sphere model [10]. Therefore, it is crucial to evaluate the chloride diffusion coefficient of mature fly ash cement paste and to quantify the primary influential factors for the durability assessment and design of cementitious materials.

In experimental investigations, Page et al. [11] designed a measuring device for the chloride diffusion in cement paste and found that the chloride diffusion coefficient is seldom influenced by the chloride concentration. MacDonald and Northwood [4] showed that the chloride diffusion coefficient decreased slightly as the sample thickness increased. Castellote et al. [12] demonstrated that, with the migration test, reliable results could be achieved through a greatly shortened measuring process. Tang and Nilsson [13] and Lu [14] also developed rapid tests for measuring the chloride diffusion coefficient of cementitious materials. Ampadu et al. [15] found that fly ash could greatly reduce the chloride diffusion coefficient of cement paste, and the optimum effect was achieved when the replacement ratio of fly ash was 40%. The effects of pre-drying and carbonation on the diffusion of chloride ions into cement paste were also evaluated [16]. Chalabi et al. [17] investigated the effect of the degree of saturation on chloride diffusion in cement paste and showed that a decrease in moisture content from 97% to 76% resulted in a higher chloride content. For a given relative humidity at 76%, the penetration depth of chloride ions was larger, while their binding to cement paste became lower. Ramirez-Ortiz et al. [18] used ultrasound to detect the presence and binding of chloride ions in cement paste with a water/cement ratio of 0.55 and found an exponential relationship between energy/amplitude-weighted average frequency and the amount of chloride chemically bound by cement paste. Akhavan and Rajabipour [19] measured the electrical conductivity with electrical impedance spectroscopy to characterize the diffusion coefficient of fiber-reinforced cement paste disks with cracks and showed that the diffusion coefficient was linearly related to the crack volume fraction. Millar et al. [20] investigated the accuracy of laser-induced breakdown spectroscopy in determining the total chloride content in cement paste. In theoretical analyses, Pivonka et al. [21] modeled cement paste as solid spheres dispersed in a pore solution medium and expressed the chloride diffusion coefficient in an analytical manner. Zheng and Zhou [22] presented an effective medium method (EMM) for the chloride diffusion coefficient of two-phase cement paste. In this approach, the Hashin–Shtrikman bounds were satisfied and the typical behaviors close to the percolation threshold were taken care of. With a digital image-based model, Garboczi and Bentz [23] investigated the chloride diffusion characteristics of cement paste. Masi et al. [24] represented the pore space with a network and showed that diffusion mainly takes place in capillary or gel pores, depending on whether the porosity was larger or smaller than the critical value. Liu et al. [25] evaluated the chloride diffusion coefficient by applying a random walk algorithm to the constructed cement paste. Ma et al. [26] estimated the chloride diffusion coefficient of cement paste with a two-scale scheme. Du et al. [27] studied the effect of external mechanical loading on the chloride diffusion coefficient of cement paste with a two-phase sphere model. They showed that the chloride diffusion coefficient decreased with the increase of the compressive volumetric strain but increased with an increasing tensile volumetric strain. Yang et al. [28] proposed a multiscale modeling technique to analyze the transport of chloride ions in cement paste and demonstrated that the contribution of the Stern layer to ion transport was not negligible for pores with a diameter less than 10 nm. Based on the reconstructed microstructure of hardened cement paste, Carrara et al. [29] used a modified Fick’s law to simulate the chloride diffusion coefficient and verified the numerical method with experimental results obtained from the literature. Damrongwiriyanupap et al. [30] employ an advanced self-consistent homogenization theory to derive an analytical relationship between the porosity, the diffusion coefficient of the pore solution, and the diffusion coefficient of cement paste. The validity of the relationship was verified with experimental results. From the above literature review, it can be seen that there are two limitations in the current studies. First, most experiments on chloride ion diffusion in cement paste were conducted at an age of 28 days or a few months, and there is a lack of experimental data on the long-term transport properties. Second, there are few analytical or numerical methods reported in the literature for evaluating the chloride diffusion coefficient of fly ash cement paste. Therefore, it is necessary to study the transport properties of mature fly ash cement paste experimentally and theoretically.

The intention of this paper is to propose an EMM for the chloride diffusion coefficient of fly ash cement paste. With fly ash cement paste modeled as a two-phase composite material, the percolation theory is incorporated into the EMM to deduce the chloride diffusion coefficient. Finally, a chloride diffusion test of fly ash cement paste is conducted to investigate the effects of the water/binder ratio, the fly ash content, and the curing age on the chloride diffusion coefficient and to verify the validity of the proposed EMM.

## 2. Numerical Estimate for Degrees of Hydration of Cement and Fly Ash

### 2.1. Degree of Hydration of Cement

The hydration of cement is traditionally assumed to proceed through a dissolution and precipitation process. According to Parrot and Killoh [31], the rate of hydration R_i,t_ of a particular clinker phase in cement is expressed as the following three equations:

(1) $$R_{i,t}=\frac{K_{1}}{N_{1}}(1-\alpha_{i,t})[-\ln(1-\alpha_{i,t}{)]}^{1-N_{1}}\;\mathrm{for}\;\mathrm{nucleation}\;\mathrm{and}\;\mathrm{growth},$$

(2) $$R_{i,t}=\frac{K_{2}{(1-\alpha_{i,t})}^{2/3}}{1-{(1-\alpha_{i,t})}^{1/3}}\;\mathrm{for}\;\mathrm{diffusion},\;\mathrm{and}$$

(3) $$R_{i,t}=K_{3}{(1-\alpha_{i,t})}^{N_{3}}\;\mathrm{for}\;\mathrm{formation}\;\mathrm{of}\;\mathrm{hydration}\;\mathrm{shell},$$

where α_i,t_ is the degree of hydration of clinker phase i (C_3_S, C_2_S, C_3_A, and C_4_AF) at time t; K_1_, N_1_, K_2_, K_3_, and N_3_ are empirical constants as listed in Table 1 [31]; and the lowest value of R_i,t_ at time t calculated from Equations (1)–(3) is selected to evaluate the instantaneous degree of hydration. Thus, the degree of hydration of cement α_c_ is taken as the weighted average of the degrees of hydration of the clinker phases.

### 2.2. Degree of Hydration of Fly Ash

In estimating the degree of hydration of fly ash α_f_, a unit volume of fly ash cement paste is considered, which includes c_0_ g of cement, f_0_ g of fly ash, and w_0_ g of water. The densities of cement, fly ash, and water are denoted by ρ_c_, ρ_f_, and ρ_w_, respectively. According to Wang and Lee [32], α_f_ is determined from the following equation:

(4) $$\frac{d\alpha_{f}}{\mathrm{dt}}=\frac{{3m}_{\mathrm{CH}}\left(t\right)}{f_{0}v_{f}r_{f0}\rho_{f}{[(1/k}_{\mathrm{df}}-r_{f0}{/D}_{\mathrm{ef}})+{(r}_{f0}{/D}_{\mathrm{ef}})(1-\alpha_{f}{)}^{-1/3}+{(1/k}_{\mathrm{rf}})(1-\alpha_{f}{)}^{-2/3}]}.$$

In Equation (4), m_CH_(t) is the CH mass, v_f_ is the stoichiometric mass ratio of CH to fly ash, r_f0_ is the radius of fly ash particles before hydration, k_rf_ is the reaction rate coefficient, and k_df_ and D_ef_ are defined as

(5) $$k_{\mathrm{df}}=\frac{B_{f}}{\alpha_{f}^{1.5}}+C_{f}{{(r}_{f0}-r_{\mathrm{ft}})}^{4}\;\mathrm{and}$$

(6) $$D_{\mathrm{ef}}=D_{\mathrm{ef}0}{\ln(1/\alpha }_{f}),$$

where D_ef0_ is the initial diffusion coefficient, r_ft_ is the radius of fly ash particles at time t, and B_f_ and C_f_ are two coefficients. The empirical coefficients included in Equations (4)–(6) are listed in Table 2. Since the hydration product CH is consumed in the second hydration reaction of fly ash, m_CH_(t) can be expressed, in terms of α_f_ and α_c_, as [33]

(7) $$m_{\mathrm{CH}}\left(t\right)=({1.32f}_{C,c}-{1.85f}_{S,c}-{2.91f}_{A,c}-{0.93f}_{F,c}{)c}_{0}\alpha_{c}-({1.85\gamma }_{S}f_{S,f}+{2.91\gamma }_{A}f_{A,f}{)f}_{0}\alpha_{f},$$

where f_C,c_, f_S,c_, f_A,c_, and f_F,c_ are the respective mass fractions of CaO, SiO_2_, Al_2_O_3_, and Fe_2_O_3_ in cement; f_S,f_ and f_A,f_ are the respective mass fractions of SiO_2_ and Al_2_O_3_ in fly ash, and the coefficients γ_S_ and γ_A_ are both taken as 0.82 [33].

### 2.3. Experimental Verification

**Figure 1:**
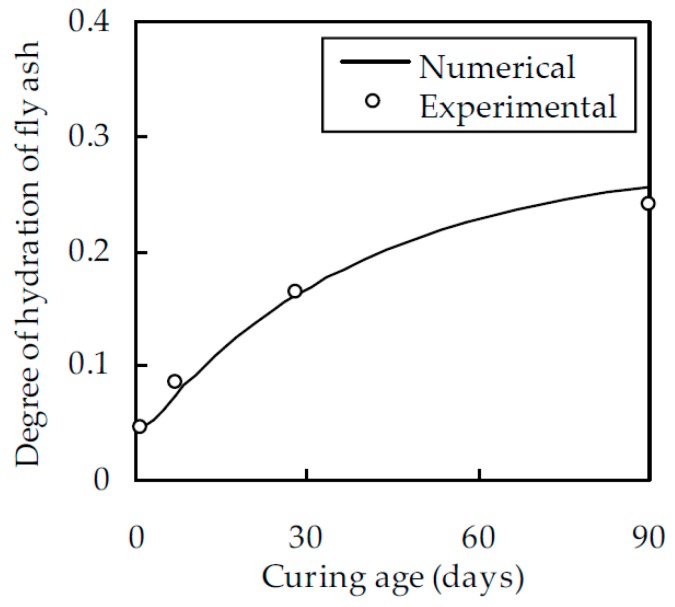
A comparison between the numerical estimate and experimental results of Haha et al. [34].

To assess the computational accuracy of the numerical estimate, the experimental results of Haha et al. [34] are considered. In their experiment, the fly ash was composed of 50% of SiO_2_ and 23.9% of Al_2_O_3_. The average particle diameter, density, and mass fraction were 14 μm, 2.74 g/cm^3^, and 35%, respectively. w/b was 0.5. α_f_ was determined with a selective dissolution technique and backscattered electron image analysis. When the curing age was 1, 7, 28, and 90 days, α_f_ was obtained as 0.045, 0.085, 0.165, and 0.24, respectively. With the same parameters as adopted in the experiment, α_f_ is estimated from Equation (4) as shown in Figure 1, which indicates a good agreement between the numerical estimate and the experimental results with a correlation coefficient of 0.996. Therefore, the degree of hydration of fly ash can be estimated with reasonable accuracy.

## 3. EMM for Chloride Diffusion Coefficient of Fly Ash Cement Paste

### 3.1. Volume Fractions of Solid Phase and Pore Space

It is well-known that Portland cement paste is a multiphase composite material, mainly composed of unhydrated cement, C–S–H, CH, and capillary pores. Gel pores are located within C–S–H and exert minor influence on chloride diffusion. By contrast, capillary pores are larger and play a major role in penetrating chloride ions into cement paste. When fly ash incorporates into cement, it reacts with CH to form additional gel pores. Therefore, as far as the diffusion of chloride ions in fly ash cement paste is concerned, it can be simplified as a two-phase medium: solid phase and pore space. According to Hansen [35] and Wang and Lee [32], at complete hydration, 1 g of cement will consume 0.36 cm^3^ of chemically bound water and form 0.19 cm^3^ of gel water, while 1 g of fly ash will consume 0.10 g of chemically bound water and form 0.15 g of gel water. Since the specific volumes of chemically bound water and gel water are 0.75 and 1 cm^3^/g, respectively, the volume fractions of gel (f_gel_) and capillary (f_cap_) pores are given by

(8) $$f_{\mathrm{gel}}=\frac{{0.19c}_{0}\alpha_{c}+{0.15f}_{0}\alpha_{f}}{c_{0}/\rho_{c}+f_{0}/\rho_{f}+w_{0}/\rho_{w}}\;\mathrm{and}$$

(9) $$f_{\mathrm{cap}}=\frac{w_{0}/\rho_{w}-{0.36c}_{0}\alpha_{c}-{0.075f}_{0}\alpha_{f}}{c_{0}/\rho_{c}+f_{0}/\rho_{f}+w_{0}/\rho_{w}}.$$

Thus, the volume fractions of the pore space (f_p_) and the solid phase (f_s_) are

$$f_{p}=f_{\mathrm{gel}}+f_{\mathrm{cap}}$$

(10) $$=\frac{w_{0}/\rho_{w}-{0.17c}_{0}\alpha_{c}+{0.075f}_{0}\alpha_{f}}{c_{0}/\rho_{c}+f_{0}/\rho_{f}+w_{0}/\rho_{w}}\;\mathrm{and}$$

$$f_{s}=1-f_{p}$$

(11) $$=\frac{c_{0}(1/\rho_{c}+{0.17\alpha }_{c})+f_{0}(1/\rho_{f}-{0.075\alpha }_{f})}{c_{0}/\rho_{c}+f_{0}/\rho_{f}+w_{0}/\rho_{w}}.$$

**Figure 2:**
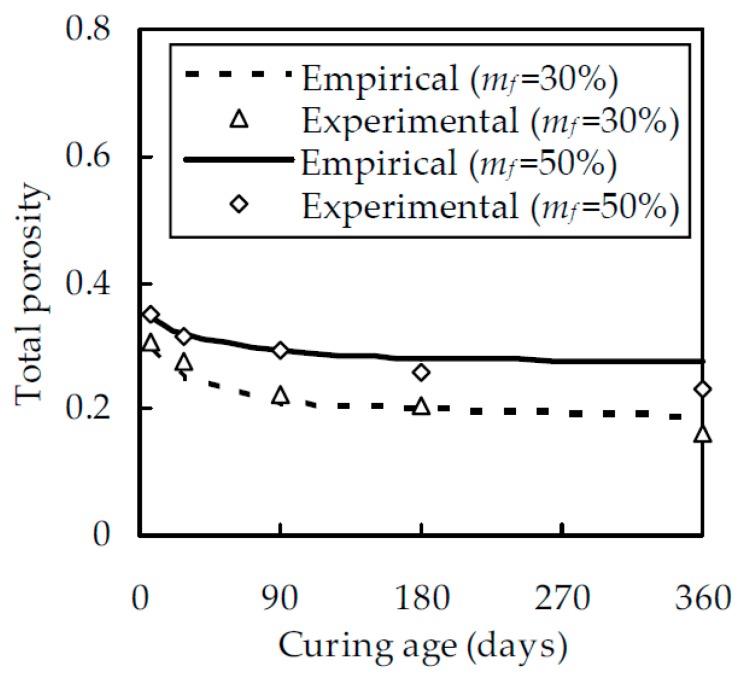
A comparison between the empirical formula and experimental results of Yu [36].

To verify the validity of the empirical formulae, the experimental results of Yu [36] are considered. In his test, w/b was 0.4 and m_f_ were 30% and 50%, respectively. The porosity was measured at curing ages of 7, 28, 90, 180, and 360 days as shown in Figure 2, which shows that the empirical formula agrees well with the experimental results. The correlation coefficient between them is 0.952 and 0.966 for a given fly ash content m_f_ at 30% and 50% by mass, respectively. Therefore, the porosity of fly ash cement paste can be estimated from the empirical formula with reasonable accuracy.

### 3.2. Chloride Diffusion Coefficient of Fly Ash Cement Paste

In evaluating the chloride diffusion coefficient, it is assumed in this paper that two-phase fly ash cement paste is an isotropic material and that gel and capillary pores are spheroids with aspect ratio μ. According to the EMM theory, the chloride diffusion coefficient D_fc_ of fly ash cement paste can be expressed as [37]

(12) $${9f}_{s}\frac{D_{s}-D_{\mathrm{fc}}}{{2D}_{\mathrm{fc}}+D_{s}}+f_{p}\left[\frac{D_{p}-D_{\mathrm{fc}}}{D_{\mathrm{fc}}+{\lambda (D}_{p}-D_{\mathrm{fc}})}+4\frac{D_{p}-D_{\mathrm{fc}}}{{2D}_{\mathrm{fc}}+(1-{\lambda )(D}_{p}-D_{\mathrm{fc}})}\right]=0,$$

where D_p_ and D_s_ are the chloride diffusion coefficients of the pore solution and the solid phase, respectively, and the depolarization factor λ is defined in terms of μ as [37]

(13) $$\lambda =\{\begin{array}{l} \frac{1}{1-\mu^{2}}\left[1-\frac{\mu }{2\sqrt{\mu^{2}-1}}\ln\left(\frac{\mu +\sqrt{\mu^{2}-1}}{\mu -\sqrt{\mu^{2}-1}}\right)\right],\;\mu >1\\ \frac{1}{3},\;\mu =1\\ \frac{1}{1-\mu^{2}}\left[1-\frac{\mu }{\sqrt{1-\mu^{2}}}\tan^{-1}\left(\frac{\sqrt{1-\mu^{2}}}{\mu }\right)\right],\;\mu <1 \end{array}.$$

Compared with the pore solution, the solid phase is almost impermeable to chloride ions, and therefore, D_s_ can be set to be zero. Thus, Equation (12) reduces to

(14) $$\frac{D_{p}-D_{\mathrm{fc}}}{D_{\mathrm{fc}}+{\lambda (D}_{p}-D_{\mathrm{fc}})}+4\frac{D_{p}-D_{\mathrm{fc}}}{{2D}_{\mathrm{fc}}+(1-{\lambda )(D}_{p}-D_{\mathrm{fc}})}=\frac{{9f}_{s}}{{2f}_{p}}.$$

After cement and fly ash are mixed with water, capillary pores in fly ash cement paste are percolated before the initial setting time. After the initial setting time, the hydration products and gel pores become a percolating network spanning fly ash cement paste. Therefore, the pore space is a percolating phase with zero percolation threshold. It should be pointed out that Equation (14) is not applicable for a percolating pore space and breaks down for fly ash cement paste. Therefore, it is necessary to modify Equation (14) by taking into account the percolation characteristics close to the percolation threshold. According to the percolation theory [38], the chloride diffusion coefficient of fly ash cement paste near zero percolation threshold is of the form

(15) $$D_{\mathrm{fc}}=D_{p}f_{p}^{n},$$

where n is a percolation exponent. In view of Eqution (15), Equation (14) can be modified as

(16) $$\frac{D_{p}^{1/n}-D_{\mathrm{fc}}^{1/n}}{D_{\mathrm{fc}}^{1/n}+{\lambda (D}_{p}^{1/n}-D_{\mathrm{fc}}^{1/n})}+4\frac{D_{p}^{1/n}-D_{\mathrm{fc}}^{1/n}}{{2D}_{\mathrm{fc}}^{1/n}+(1-{\lambda )(D}_{p}^{1/n}-D_{\mathrm{fc}}^{1/n})}=\frac{{9f}_{s}}{{2f}_{p}}.$$

Since D_p_ > 0, Equation (16) reduces to

(17) $$\frac{1-{\left(D_{\mathrm{fc}}{/D}_{p}\right)}^{1/n}}{{\left(D_{\mathrm{fc}}{/D}_{p}\right)}^{1/n}+\lambda [1-{\left(D_{\mathrm{fc}}{/D}_{p}\right)}^{1/n}]}+4\frac{1-{\left(D_{\mathrm{fc}}{/D}_{p}\right)}^{1/n}}{2{\left(D_{\mathrm{fc}}{/D}_{p}\right)}^{1/n}+(1-\lambda )[1-{\left(D_{\mathrm{fc}}{/D}_{p}\right)}^{1/n}]}=\frac{{9f}_{s}}{{2f}_{p}}.$$

Solving Equation (17) for (D_fc_/D_p_)^1/n^ gives

(18) $$\frac{D_{\mathrm{fc}}^{1/n}}{D_{p}^{1/n}}=\frac{4-k+(k-6)\lambda -{2k\lambda }^{2}+\sqrt{36-4k+k^{2}+(12k-{6k}^{2})\lambda +{9k}^{2}\lambda^{2}}}{10+2k-6\lambda -{2k\lambda }^{2}},$$

where k is defined as

(19) $$k=\frac{{9f}_{s}}{{2f}_{p}}.$$

Thus, D_fc_ can be evaluated from Equation (18).

## 4. Experimental Verification and Discussions

### 4.1. Materials, Methods, and Results

To verify the EMM for the chloride diffusion coefficient of fly ash cement paste, a chloride diffusion text was conducted in this study. In the test, fly ash cement paste was made with an ordinary Portland cement of grade P.O 42.5 and a fly ash of grade II. The apparent densities of the cement and fly ash were 3150 and 2300 kg/m^3^, respectively, and the chemical compositions are listed in Table 3. The water/binder ratio w/b was selected as 0.4, 0.5, and 0.6. For each w/b, one mix was cast with the cement, while the other three mixes were cast by replacing m_f_ = 10%, 20%, and 30% by mass of the cement with the fly ash. Tap water with a pH value of 6.5 was used for mixing and curing the fly ash cement paste.

**Figure 3:**
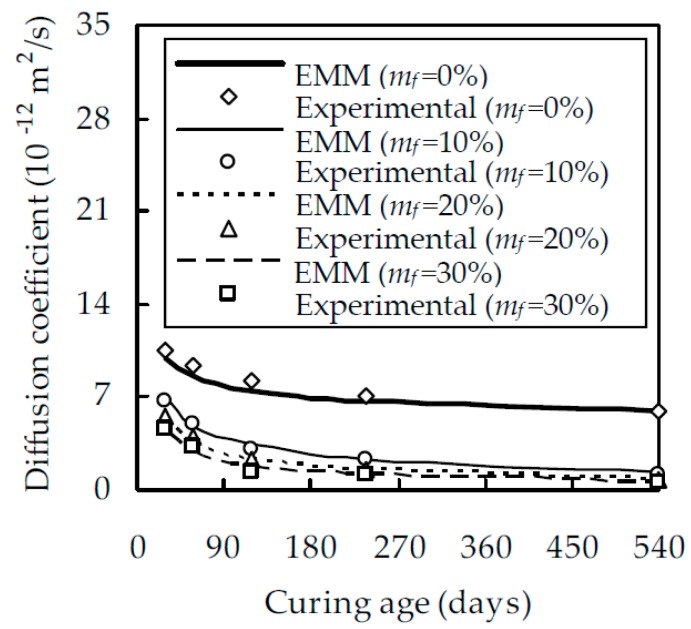
The relationship between D_fc_ and t for fly ash cement paste with w/b of 0.5.

**Figure 4:**
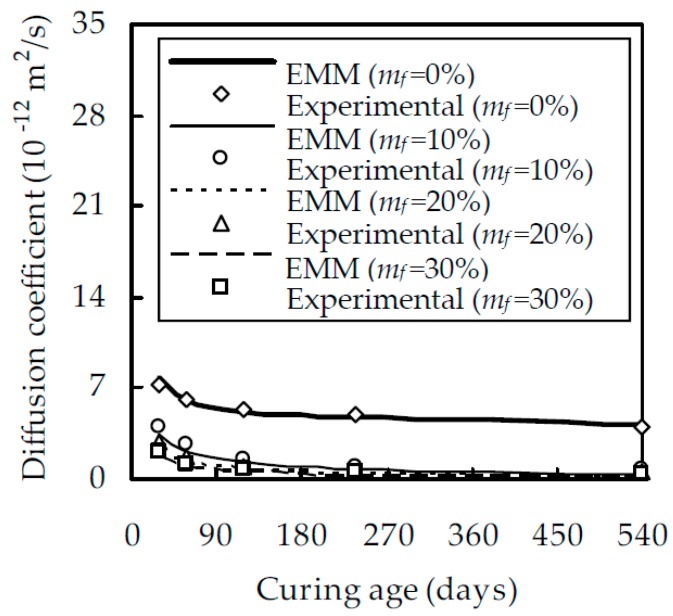
Relationship between D_fc_ and t for fly ash cement paste with w/b of 0.4.

**Figure 5:**
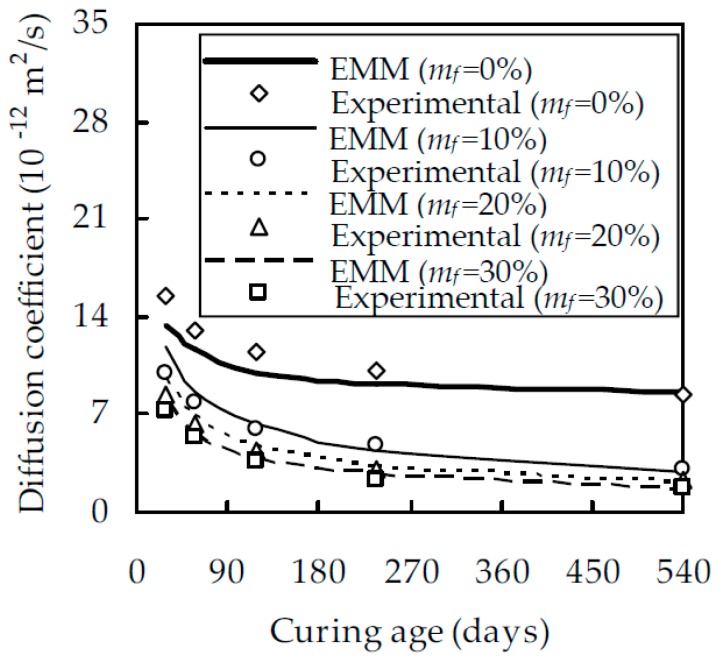
Relationship between D_fc_ and t for fly ash cement paste with w/b of 0.6.

Compared with concrete, cement paste is more uniform and the measured chloride diffusion coefficients for the same mix have a smaller scatter. Therefore, for each mix, three identical cylindrical specimens 100 mm thick and 100 mm in diameter were made to measure the average chloride diffusion coefficient. After 24 h of casting, the demolded specimens were cured in the laboratory. The temperature and relative humidity were 20 ± 1 °C and 95 ± 1%, respectively. At a given curing age, the specimens were taken out from the curing room and the mid-part with a thickness of 50 mm was cut off. The chloride diffusion coefficient was measured with the accelerated method proposed by Lu [14], as shown in Figure 3, Figure 4 and Figure 5.

When w/b is 0.5, the relationship between the chloride diffusion coefficient D_fc_ and the curing age t is shown in Figure 3 for different fly ash contents m_f_, which indicates that, at a given t, D_fc_ decreases with the increase of m_f_. The reason for this is that, within a reasonable value of m_f_, fly ash particles uniformly distribute in the cement paste, fill in the gel and capillary pores, and improve the pore structure. When m_f_ increases from 0% to 10%, D_fc_ decreases evidently. However, when m_f_ is larger than 10%, the effect of fly ash on D_fc_ reduces gradually. For a given t at 28, 58, 118, 238, and 540 days, D_fc_ at m_f_ = 10% is smaller than that at m_f_ = 0% by 36.1%, 46.7%, 63.1%, 66.3%, and 79.4%, while D_fc_ at m_f_ = 30% is smaller than that at m_f_ = 10% by 32.9%, 43.4%, 53.8%, 62.3%, and 49.6%, respectively. Figure 3 also demonstrates that, for fly ash cement pastes with m_f_ = 20% and 30%, their chloride diffusion coefficients are very close, especially at the later ages of curing, which is consistent with the results of Oh and Jang [39]. It should also be pointed out that, although D_fc_ decreases with increasing m_f_, an excessively large m_f_ will appreciably reduce the degree of hydration of fly ash and second hydration products. As expected, D_fc_ decreases with an increase in curing age t. When m_f_ is 0%, 10%, 20%, and 30%, D_fc_ at 540 days is smaller than that at 28 days by 43.1%, 81.7%, 84.9%, and 86.2%, respectively. This is due to the fact that the pozzolanic reaction of fly ash mainly takes place after 28 days [40]. With the pozzolanic reaction, a great quantity of second C–S–H is produced, the size of large capillary pores is reduced, and the resistance to chloride ingress is enhanced. When w/b is equal to 0.4 and 0.6, the relationship between D_fc_ and t is shown in Figure 4 and Figure 5, respectively. A comparison of Figure 3, Figure 4 and Figure 5 shows that, for a given m_f_ and t, D_fc_ decreases with a decreasing w/b. When t is 540 days and w/b decreases from 0.6 to 0.4, D_fc_ decreases by 32.4%, 55.9%, 78.7%, and 81.7% for a given m_f_ at 0%, 10%, 20%, and 30%, respectively. This is attributed to the fact that a larger w/b will result in a larger porosity and a smaller resistance to chloride ingress.

### 4.2. Experimental Verification

As can be seen from Equations (13) and (18), μ, D_p_ and n need to be determined to evaluate D_fc_. From the experimental calibration, μ was obtained as 3.263 [41], which is in excellent agreement with that estimated from the relationship between the critical volume fraction of pores and the aspect ratio [42]. It is appreciated that D_p_ is related to the viscosity of pore solution [21], temperature, and the microstructure of cement paste [43] and is, at present, difficult to measure in the laboratory. As an indirect method, Pivonka et al. [21] and Damrongwiriyanupap et al. [30] obtained through experimental calibration the values of D_p_ as 1.07 × 10^−10^ and 1.476 × 10^−10^ m^2^/s, respectively. Since the two values are very close, D_p_ is taken as 1.07 × 10^−10^ m^2^/s in this paper. Up to now, the percolation exponent n is still an open issue. For three-dimensional invariable porous media, it has been widely accepted that n is equal to 2 [44]. Wong et al. [45] demonstrated that n is between 1.5 and 2 for three-dimensional porous media. For sedimentary rocks with different microstructures, the value of n calibrated from Archie’s law varies from 1.5 to 4 [46]. Oh and Jang [39] showed from experimental results that n is equal to 2.7 for hardened cement paste and increases to 4.5 in the presence of fly ash or slag. They attributed the increase of n to the improvement of the pore structure caused by mineral admixtures. From the above discussions, it is seen that experimental calibration is possibly the only feasible method. For this purpose, the experimental results obtained in this study for w/b = 0.5 shown in Figure 3 are considered. With these experimental date, n is back-calculated and regressed, in terms of m_f_, α_c_ and α_f_, as

(20) $$n=({15.13m}_{f}+{2.829)[m}_{f}\alpha_{f}+(1-m_{f})\alpha_{c}]-\frac{{76.37m}_{f}+3.914}{{37.39m}_{f}^{1.8}+6.614}.$$

The numerical results show that the correlation coefficient between Equation (20) and the back-calculated values of n is equal to 0.890.

With μ, D_p_, and n known, Equation (18) can be used to evaluate the chloride diffusion coefficient of fly ash cement paste. For w/b = 0.4 and 0.6, the result are shown in Figure 4 and Figure 5, respectively. It is seen from Figure 4 and Figure 5 that the analytical estimate is in good agreement with the experimental results. When m_f_ = 0%, 10%, 20%, and 30%, the correlation coefficient between them are 0.996, 0.984, 0.987, and 0.984 for w/b = 0.4 and 0.997, 0.959, 0.973, and 0.972 for w/b = 0.6, respectively. Therefore, it is concluded that the EMM can estimate the chloride diffusion coefficient of fly ash cement paste with reasonable accuracy.

## 5. Conclusions

An EMM was proposed to evaluate the chloride diffusion coefficient of fly ash cement paste, and a chloride diffusion test was conducted to investigate the transport properties of fly ash cement paste. The conclusions of this study are summarized in the following points:

1. Fly ash cement paste was modeled as a two-phase material, and the volume fraction of each phase constituent was formulated in terms of the water/binder ratio and the numerically estimated degrees of hydration of cement and fly ash.
2. By considering the percolation characteristics close to the percolation threshold, the percolation theory was incorporated into the EMM and the chloride diffusion coefficient of fly ash cement paste was derived and verified with experimental results.
3. The experimental results show that, when w/b was equal to 0.5 and t was 28 and 540 days, D_fc_ at m_f_ = 30% was smaller than that at m_f_ = 0% by 55.7% and 90.0%, respectively. When w/b was equal to 0.5 and m_f_ was 0% and 30%, D_fc_ at 540 days was smaller than that at 28 days by 43.1% and 86.2%, respectively. When t was 540 days and w/b decreased from 0.6 to 0.4 D_fc_ decreased by 32.4% and 81.7% for m_f_ = 0% and 30%, respectively.

## Figures and Tables

**Table 1:** The kinetic parameters of clinker phases at different hydration stages.

Kinetic Parameter	Clinker Phase			
	C_3_S	C_2_S	C_3_A	C_4_AF
K_1_	1.5	0.5	1.0	0.37
N_1_	0.7	1.0	0.85	0.7
K_2_	3.3	5.0	3.2	3.7
K_3_	0.05	0.006	0.04	0.015
N_3_	1.1	0.2	1.0	0.4

**Table 2:** The parameters in the hydration model of fly ash.

B_f_ (cm/h)	C_f_ (cm/(cm^4^·h))	k_rf_ (cm/h)	D_ef0_ (cm^2^/h)
2.51 × 10^−9^	1.00 × 10^15^	1.71 × 10^−6^	8.58 × 10^−8^

**Table 3:** Chemical compositions of fly ash and cement.

Material	CaO (%)	SiO_2_ (%)	Al_2_O_3_ (%)	Fe_2_O_3_ (%)	MgO (%)	SO_3_ (%)	K_2_O (%)	Na_2_O (%)	Loss of Ignition (%)
Fly ash	5.40	47.00	31.30	4.40	0.49	0.77	0.90	0.78	3.22
Cement	64.40	20.36	4.96	3.17	2.09	1.98	0.64	0.14	1.27
